# Antigenic Relationships among Human Pathogenic *Orientia tsutsugamushi* Isolates from Thailand

**DOI:** 10.1371/journal.pntd.0004723

**Published:** 2016-06-01

**Authors:** Sarah L. James, Stuart D. Blacksell, Pruksa Nawtaisong, Ampai Tanganuchitcharnchai, Derek J. Smith, Nicholas P. J. Day, Daniel H. Paris

**Affiliations:** 1 Center for Pathogen Evolution, Department of Zoology, University of Cambridge, Cambridge, United Kingdom; 2 World Health Organization (WHO) Collaborating Center for Modeling, Evolution, and Control of Emerging Infectious Diseases, Cambridge, United Kingdom; 3 Mahidol-Oxford Tropical Medicine Research Unit, Faculty of Tropical Medicine, Mahidol University, Bangkok, Thailand; 4 Centre for Tropical Medicine & Global Health, Nuffield Department of Medicine, Oxford, United Kingdom; 5 Department of Viroscience, Erasmus Medical Center, Rotterdam, Netherlands; University of Otago, NEW ZEALAND

## Abstract

**Background:**

Scrub typhus is a common cause of undiagnosed febrile illness in certain tropical regions, but can be easily treated with antibiotics. The causative agent, *Orientia tsutsugamushi*, is antigenically variable which complicates diagnosis and efforts towards vaccine development.

**Methodology/Principal Findings:**

This study aimed to dissect the antigenic and genetic relatedness of *O*. *tsutsugamushi* strains and investigate sero-diagnostic reactivities by titrating individual patient sera against their *O*. *tsutsugamushi* isolates (whole-cell antigen preparation), in homologous and heterologous serum-isolate pairs from the same endemic region in NE Thailand. The indirect immunofluorescence assay was used to titrate *Orientia tsutsugamushi* isolates and human sera, and a mathematical technique, antigenic cartography, was applied to these data to visualise the antigenic differences and cross-reactivity between strains and sera. No functional or antigen-specific analyses were performed. The antigenic variation found in clinical isolates was much less pronounced than the genetic differences found in the 56kDa type-specific antigen genes. The Karp-like sera were more broadly reactive than the Gilliam-like sera.

**Conclusions/Significance:**

Antigenic cartography worked well with scrub typhus indirect immunofluorescence titres. The data from humoral responses suggest that a Karp-like strain would provide broader antibody cross-reactivity than a Gilliam-like strain. Although previous exposure to *O*. *tsutsugamushi* could not be ruled out, scrub typhus patient serum antibody responses were characterised by strong homologous, but weak heterologous antibody titres, with little evidence for cross-reactivity by Gilliam-like sera, but a broader response from some Karp-like sera. This work highlights the importance of antigenic variation in *O*. *tsutsugamushi* diagnosis and determination of new serotypes.

## Author Summary

Scrub Typhus is a common and potentially severe febrile illness in certain tropical regions in Asia. This infection is treatable with specific antibiotics if diagnosed correctly, but there is no effective vaccine available at present. The bacterium causing this disease is called *Orientia tsutsugamushi*; it is transmitted by small mites and it has variable surface proteins, which make diagnosis and vaccine development difficult. In this study, we tested how well the antibodies in the blood of patients with scrub typhus recognise different strains of bacteria, and used these results to create a map of the relationships between the bacteria and sera. From examining this map we can see that some sera have activity against a wider range of bacteria than others. These methods and findings will help with selecting bacteria strains and evaluating immune responses, which will potentially help us to improve diagnosis and vaccine development.

## Introduction

*Orientia tsutsugamushi* is an antigenically variable pathogen. This obligate intracellular bacterium causes scrub typhus, a common tropical rickettsial febrile illness endemic across much of the Asia-Pacific region [1–5]. *O*. *tsutsugamushi* is vertically maintained in mites of the *Trombiculidae* family and transmitted to humans by the bite of the larval stage, called chiggers [6]. Scrub typhus is the leading cause of treatable febrile illness and endemic in many parts across Asia. Despite its easily treatable nature, scrub typhus is difficult to diagnose and no vaccine is currently available. Although antibiotic therapy with either doxycycline or azithromycin can achieve an effective cure, treatment does not affect incidence rates, as humans are dead-end hosts [7]. Further, it was shown that protective immunity to a homologous strain can last several years, but heterologous protection in treated patients and natural disease survivors can last for a few months only. This short-lived heterologous, but intermediate to long-lived homologous immunity results in a high recurrence rate of disease, which is further complicated by the broad antigenic heterogeneity of strains [6,8,9].

Historically, *O*. *tsutsugamushi* (formerly *Rickettsia tsutsugamushi*) was classified into antigenic groups on the basis of their sero-reactivity against prototype strains (i.e. Karp, Kato, and Gilliam). Since the 1940s, the discovery of the antigenic heterogeneity of *O*. *tsutsugamushi* strains has posed a real obstacle for all progress regarding strain classification, diagnostic and vaccine development and epidemiological studies of scrub typhus. Numerous functional cross-reactivity and cross-vaccination studies have contributed towards the characterization of Orientia immunogens and their strain-specific or group-specific serological properties [10–12]. Unfortunately this work has not led to any translational output towards an improved classification scheme or identification of broadly cross-protective antigens.

Antigenic cartography is a computational tool that is applied to assays of cross-reactivity, and can transform datasets of serological titres into an antigenic map. These maps enable a quantitative visualization of relevant antigenic variation among the pathogens [13]. This methodology has been applied successfully to viral diseases, mainly influenza, and also dengue virus, foot and mouth disease virus, lyssavirus, flavivirus and enterovirus 71, but not to intracellular bacteria [14–18]. The basis of antigenic cartography relies on the measurement of titres derived from haemagglutinin inhibition, plaque inhibition, immunofluorescence or neutralization assays producing an endpoint titre that quantitates the neutralizing or diagnostic capacity of the antibody and feeds into the antigenic map. Unfortunately, plaque inhibition assays are not useful due to the fastidious nature of *O*. *tsutsugamushi* and the fact that that most strains do not produce a reliable cytopathic effect (CPE)—although some strains can produce CPE, but only after multiple passages in cell culture. Neutralisation assays would be more appropriate, but antibodies that target the highly strain-specific neutralizing epitopes of *O*. *tsutsugamushi* do not sufficiently represent the humoral immune response [19–23]. Hence we opted to use binding endpoint titres (BETs)—determined by 2-fold serial serum dilutions titrated onto specially produced single-strain IFA slides—as representative titres to feed into the analysis for antigenic mapping.

The 56-kDa type-specific antigen (TSA) located on the outer membrane surface of *O*. *tsutsugamushi* is the major immunogen and responsible for eliciting neutralizing antibodies [19–23]. Similar to the lyssavirus trimeric glycoprotein or the influenza haemagglutinin, the 56-kDa TSA is a highly variable surface antigen involved in cell binding and entry and target for neutralizing antibodies [16,22–24].

The gene encoding the 56-kDa TSA has an ORF of approximately 1,600 bp length, and with its four hypervariable regions contributes substantially to the high diversity among Orientia strains [20,25]. This has hampered the progress on diagnostic test development and candidate vaccine selection [25–27]. The majority of anti-*Orientia* antibodies of acute and convalescent patient serum contain anti-56-kDa TSA antibodies [20,21]. There are no functional studies that have investigated 56-kDa TSA associated immunoglobulin isotypes and possible effector function that affect immune protection, like antibody cytotoxicity.

In an effort to understand more completely the antigenic and genetic relatedness of *O*. *tsutsugamushi* strains and shed light on sero-diagnostic obstacles, we titrated patient sera against a collection of Thai isolates (including isolate—serum pairs from individual infections) from the same endemic region in NE Thailand and performed antigenic cartography. The antigenic map approach allows us to evaluate if the historically defined Orientia serotypes actually match the observed antigenic clusters on the map, if any antigenic subtypes or clusters exist within these serotypes and how quantitatively different these clusters are.

## Methods

### Patient specimens and isolates

Whole blood samples were collected from scrub typhus patients in Udon Thani (535 km Northeast of Bangkok) and Tak (512 km Northwest of Bangkok) provinces between September 2003 and August 2005. Twenty-one *O*. *tsutsugamushi* isolates were grown *in vitro* using the method described previously (Table 1) [28]. Seventeen isolates (19/23; 83%) were from Udon Thani patients (termed UT samples) and four (4/23; 17%) isolates were from Tak patients (FPW samples). Admission and convalescent (where possible) serum was also collected from each patient from which there was an *O*. *tsutsugamushi* isolate, and two additional serum samples were analysed (sera n = 23).

**Table 1:** Description of Thai *O*. *tsutsugamushi* isolates of human origin used in this study.

Isolate	Month/Year isolated	District	Province /Prefecture	Gene length (bp)	GenBank Accession No.	Genotype strain
UT76	09/2003	Muang	Udon Thani	1611	EF213078	Karp
UT125	10/2003	Muang	Udon Thani	1596	EF213096	Gilliam
UT144	06/2004	Muang	Udon Thani	1596	EF213091	Gilliam
UT150	06/2004	Muang	Udon Thani	1611	EF213086	Karp
UT167	06/2004	Phen	Udon Thani	1611	EF213080	Karp
UT169	06/2004	Muang	Udon Thani	1608	EF213092	Karp
UT176	07/2004	Ban Phu	Udon Thani	1602	EF213081	Karp
UT177	07/2004	Muang	Udon Thani	1605	EF213084	Karp
UT196	07/2004	Muang	Udon Thani	1596	EF213079	Gilliam
UT213	07/2004	Sang Khom	Udon Thani	1611	EF213088	Karp
UT219	07/2004	Muang	Udon Thani	1611	EF213100	Karp
UT221	08/2004	Muang	Udon Thani	1614	EF213097	Karp
UT302	08/2004	Muang	Udon Thani	1587	EF213095	TA763
UT316	10/2004	Muang	Udon Thani	1611	EF213082	Karp
FPW2016	05/2004	Pho Pra	Tak	1608	EF213085	Gilliam
FPW1038	10/2004	Mae Ramat	Tak	1593	EF213087	TA716
FPW2031	12/2004	Pho Pra	Tak	1614	EF213098	Karp
UT329	7/2005	Na Yang	Udon Thani	1596	EF213099	Gilliam
UT332	7/2005	Muang	Udon Thani	1611	EF213083	Karp
UT336	7/2005	Wang Sam	Udon Thani	1599	EF213089	Karp
UT395	7/2005	Muang	Udon Thani	1611	EF213094	Karp
FPW2049	7/2005	Pho Pra	Tak	1596	EF213093	Gilliam
UT418	8/2005	Muang	Udon Thani	1605	EF213090	Karp

Note: *Original in vitro isolation and 56 kDa genotyping data is presented elsewhere [36].*

### Ethics statement

Ethical approval was obtained from the Faculty of Tropical Medicine, Mahidol University (Tak study), the Thai Ministry of Public Health (Udon Thani study), and the Oxford Tropical Research Ethics Committee (both studies). All patients in this study provided written informed consent prior to sample collection, if minors were participants, a parent or guardian of any child participant provided written informed consent on their behalf.

### *O*. *tsutsugamushi* isolates

*Orientia* were propagated in Vero cell cultures in 25 cm^2^ tissue culture flasks under biocontainment level 3 conditions. The isolates were harvested when 100% cytopathic effect was microscopically evident, and/or serial cell scrapings reached 100% infection as determined by immunofluorescence microscopy. Whole cell lysates of *O*. *tsutsugamushi* cultures were prepared by mechanical scraping of cells from culture flasks, centrifuging the suspension at 750xg for 10 minutes, discarding the supernatant, re-suspending cells in phosphate-buffered saline (PBS), and repeated pipetting to ensure a uniform host cell lysate solution. The lysate (2ul) was spotted onto each well of 40-well Teflon-coated microscope slides, air-dried and fixed in cold acetone for 10 min. The slides were assessed for uniform distribution of the cell lysate antigen by immunofluorescence microscopy. The slide was either used immediately for further investigations or stored at -20°C until required.

### Antigenic analysis

Antigenic cross-binding analysis was carried out based on a method previously described [29,30]. The method used micro-immunofluorescence to determine antigenic relationships between *O*. *tsutsugamushi* isolates by assessing the level of patient serum binding to homologous and heterologous isolates. Patient sera were serially 2-fold diluted from 1:50 to 1:12,800 in PBS containing 2% (w/v) skim milk powder and incubated in a humidified atmosphere for 30 minutes at 37°C followed by 3 washing cycles in PBS. Anti-human IgA+IgG+IgM FITC conjugate (Jackson, USA) diluted in PBS-SMP diluent containing 0.00125% (w/v) Evans Blue counterstain was applied to all wells and incubated in a humidified atmosphere for 30 minutes at 37°C. The cells were examined by fluorescence microscopy at a magnification of 200x and the binding endpoint titre (BET) was determined as the highest dilution displaying fluorescence, and expressed as the reciprocal value (i.e. 800 for 1:800). Hence, each serum was attributed a BET. For comparisons the reciprocal median titres (RMTs) were calculated within the Karp, Gilliam and TA716 groups; the BETs of related strains within a group were divided by the homologous-paired BET with the reference strain for normalization (Table 2). Using R software, a heatmap was created based on correlations between normalised patient serum RMTs against the different isolates [31,32].

**Table 2:** Summary of homologous and heterologous titres, normalized to give a maximum titre of 100.

Patient serum (n = 23)						
	Homologous paired titres RMT (range)		Heterologous paired titres RMT (range)			
			Karp (n = 15)	Gilliam (n = 6)	TA716 (n = 1)	TA763 (n = 1)
	Karp (n = 14)	75 (3.1–100)	50 (0.8–100)	12.5 (0.4–100)	50 (12.5–100)	18.8 (6.3–100)
Patient Thai *O*. *tsutsugamushi* isolates (n = 21)	Gilliam (n = 6)	100 (100–100)	25 (0–100)	75 (6.3–100)	100 (25–100)	100 (25–100)
	TA716 (n = 1)	100 (NA)	24.9 (0.4–100)	3.1 (0.4–25)	100 (NA)	6.3 (NA)

*Abbreviations*: *NA*: *not applicable as one isolate assessed*. *RMT; the reciprocal median titres (RMTs) were calculated by dividing the binding endpoint titre (BET) of each serum sample to various isolates*, *by the BET against its homologous paired strain and reported reciprocally*. *The median RMTs were calculated per group*. *The binding endpoint titre (BET) was determined as the highest dilution displaying positive fluorescence*, *and expressed as the reciprocal value (i*.*e*. *1*,*600 for 1*:*1*,*600*, *and if the homologous paired titer was 12*,*800*, *then the RMT was 1*,*600/12*,*800 = 0*.*125 and reported as RMT 12*,*5)*.

The titers from heterologous paired samples showed that on average anti-Karp sera reacted broader against Gilliam and TA716 strains than anti-Gilliam sera reacted against the Karp-like strains. This data does not illustrate cross-protection, but rather that anti-Karp sera reacted broadly within Karp and showed more cross-reactivity to Gilliam, TA716 and TA763 clusters, while sera raised against Gilliam, remained very Gilliam-specific.

### Genotyping

The complete ORF of 56-kDa TSA gene was amplified by conventional PCR using previously described assays complemented with primers for optimal coverage [33–35]. Nucleotide sequencing was performed by Macrogen, Korea (the MegaBACE Model 1000 automated sequencer (Amersham Bioscience, UK).

### Phylogenetic analysis

Multiple gene sequence alignment was performed using Clustal X [36]. The aligned sequences of the 56-kDa TSA protein for the strains and sera were used to construct an amino acid phylogenetic tree using PhyML [37]. The LG model of substitution was used with 10 random starts and 1000 bootstrap replicates, using the both Nearest Neighbour Joining and Subtree Pruning and Regrafting. The gamma distribution parameter from estimated from the data and the equilibrium frequencies were taken from the frequencies defined by the substitution model. Both the branch lengths and substitution model parameters were optimised. The best tree and bootstrap values was plotted using R [31,38].

### Antigenic cartography

Antigenic cartography is a tool that transforms a table of antigenic data (i.e. cross-reactivity titres between strains and sera) into a map of the antigenic relationships between these strains and sera, using the mathematical technique of multi-dimensional scaling. This method was designed for the influenza virus, and typically uses sera from a primary exposure, to exclude confounding pre-existing antibody [13].

The table of titres can be considered a table of antigenic relatedness; a high titre of a particular serum against a particular isolate indicates similarity, while a low titre indicates difference. The tabularized data can mathematically be transformed into a map, such that a serum and antigen pair with a high titre has a small distance between them, and the map distances correlate inversely with the titres in the table. An initial map is generated and then the points (antigens and sera) are moved around in iterations so that the map distances match the table distances better. This is repeated with different random starting points. Thus an antigenic map represents the coordinates for all the antigens and sera used, with the distance between sera and isolates reflecting similarity. More specifically, the titre of serum j against antigen i, termed T_ij_, is transformed into antigenic distances, D_ij_, using the equation:

$${\text{D}}_{\text{ij}}={\text{b}}_{\text{ij}}\text{-}{\text{log}}_{2}({\text{T}}_{\text{ij}})$$

Where b_ij_ is log base 2 of the maximum titre against serum j. A map is generated which minimizes the error function:

$$\Sigma_{\text{ij}}({\text{D}}_{\text{ij}}\text{-}{\text{d}}_{\text{ij}})$$

Where d_ij_ is the Euclidean distance between antigen i and serum j in the map. In the case where d_ij_ thresholded titre, for example a titre of <10, the error function is altered so that this titre only contributes to the error function when d_ij_ < D_ij_-1 [13].

Maps were created with 1, 2, 3, 4 and 5 dimensions. The maps were optimised by removing a certain proportion (10%, 20%, 30%, 40% or 50%) of the data, and the ability of the map to predict the excluded titres was evaluated. These analyses were performed using lispMDS software [39]. A similar process can be performed on sequence data, treating the number of amino acid substitutions as the distance between the two antigens.

## Results

### Genetic variation

**Fig 1:**
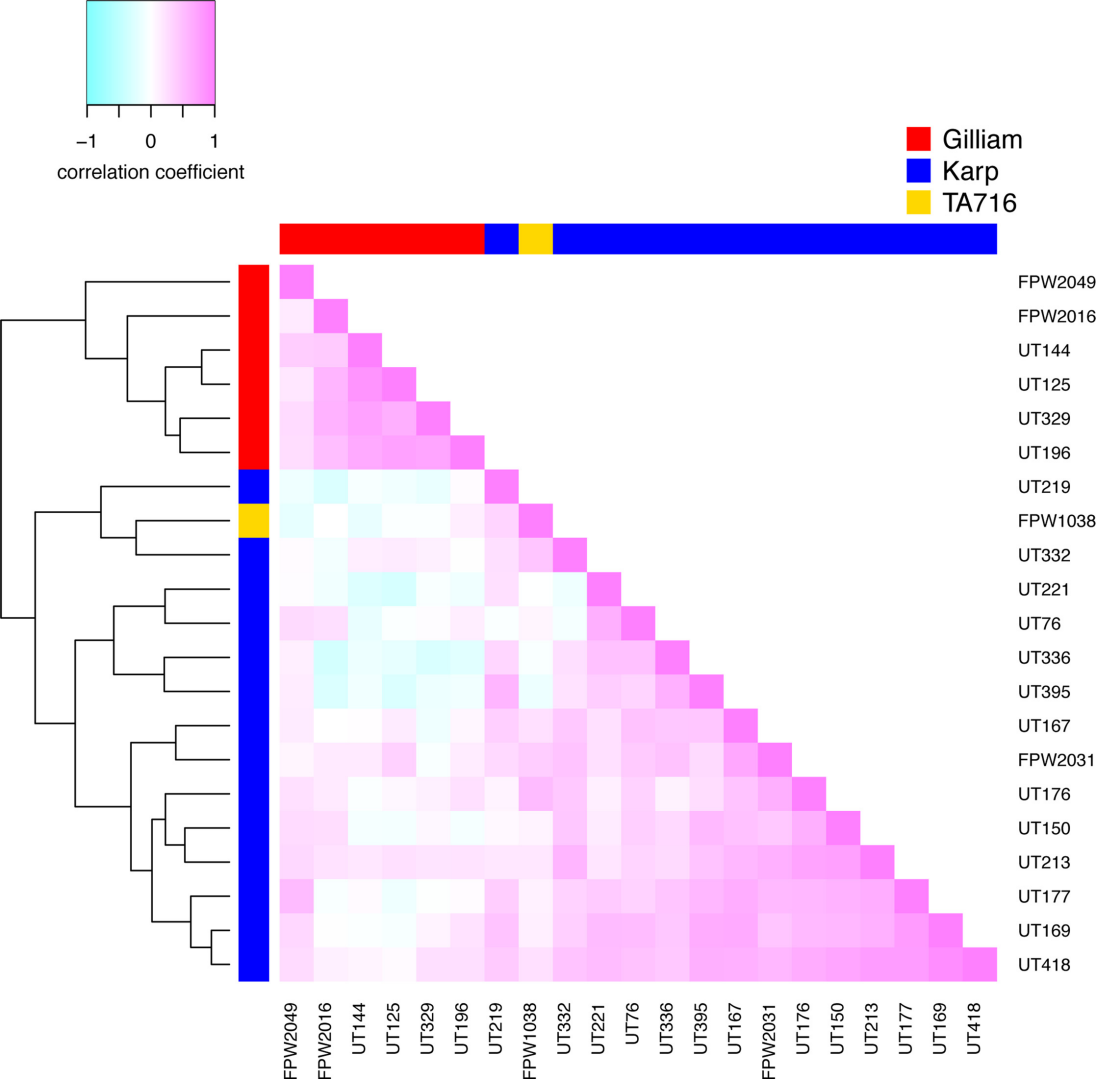
Antigenic relatedness of Thai *O*. *tsutsugamushi* isolates against homologous and heterologous sera. Heatmap of the correlation between the patient serum responses of the different isolates. The dendrogram was produced using *distance = 1-correlation*. Genetic assignment of the *O*. *tsutsugamushi* isolates based on 56 kDa gene analysis is represented by the colored bars on top and left of the heat map (Gilliam in red, Karp in blue and TA716 in yellow).

Genetic analysis of the 56-kDa TSA ORF demonstrated that the 21 Thai *O*. *tsutsugamushi* isolates and two additional sera in this study were related to Karp, Gilliam and TA716 prototype strain genotypes (Fig 1 and Table 2). Phylogenetic details have been discussed previously [35].

### Antigenic variation

**Fig 2:**
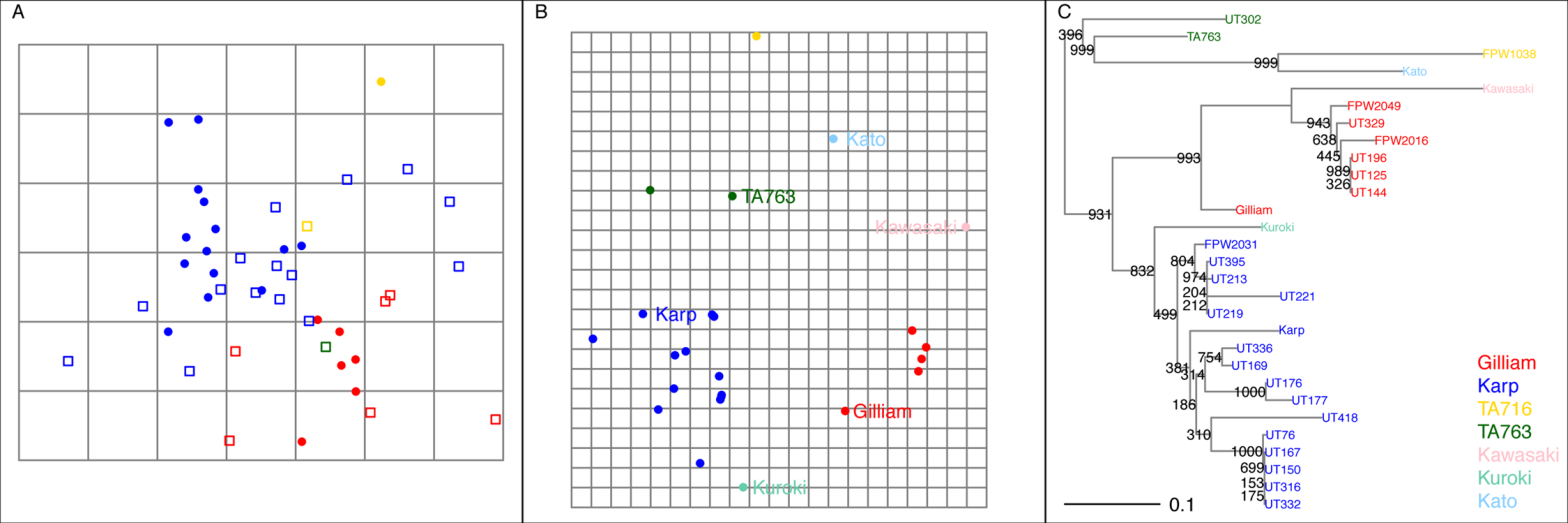
An overview of antigenic mapping and genetic variation of *O*. *tsutsugamushi* Thai isolates. Panel A: Antigenic map of indirect immunofluorescence titres in Table 3. Calculating the antigenic distance between points gives a measure of antigenic similarity allowing quantitative visualization of serological data for *O*. *tsutsugamushi*. Points close to each other are antigenically similar. Each circle represents an *O*. *tsutsugamushi* isolate, and each square represents a serum. The grid background of panel A indicates antigenic distance; the spacing between grid lines is 1 antigenic unit, corresponding to a twofold dilution of patient sera in the indirect immunofluorescence assay. The points are colored according to their genetic group, as determined in panel C. Panel B: A genetic map of the strains and sera in panel A, with additional prototype strains. The grid spacing is every 10 units of genetic distance (amino acid mutations). The genetic map was made using the same method as the antigenic map, but using the genetic distance (number of mutations) as opposed to a measure of antigenic distance. Panel C: A phylogenetic tree of the 56kDa protein amino acid sequences of *O*. *tsutsugamushi* strains and human sera used in this study, with bootstrap values on the nodes.

Homologous and heterologous reactivity of antigen and patient serum pairs demonstrated distinct groupings that corresponded to the Karp and Gilliam clusters based on 56-kDa TSA genetic analysis; the sera raised against Gilliam-like strains discriminated between Karp-like and Gilliam-like strains (Table 2). Antibody titers in homologous serum-isolate pairs were not always highest in Karp/ Karp-like samples, whereas for Gilliam and TA716 strains this was the case (Table 2). Sera raised against Karp strains reacted more weakly with corresponding homologous strains (Karp RMT 75) than the sera homologous strain pairs for Gilliam and TA716 strains (homologous RMTs each 100). However, the heterologous paired titres in the Karp group showed a greater reactivity against all other strains (Karp RMT 50; Gilliam RMT 12.5; TA716 RMT 50; TA763 RMT 18.8), than Gilliam sample pairs did (Karp RMT 25; Gilliam RMT 75; TA716 RMT 25; TA763 RMT 100). Sera from infections with Karp-like genotype demonstrated greater heterologous antigen reactivity, with broader cross-reactivity into Gilliam-like isolates (Fig 2, panel A).

### Antigenic cross-reactivity results

The dendrogram generated from correlations between normalised titres demonstrated two main antigenic groups which associated with the Karp and Gilliam genotypic strains, with the TA716-related isolate bifurcating within the Karp-like grouping (Fig 1), the heatmap, showing mainly positive correlation between normalised IFA-based BETs. However, the serum responses to the Gilliam-like isolates show negative correlations to a set of serum responses to the Karp-like isolates. Generally higher correlations, expressed as darker shades of pink (Fig 1), were found within genotypes rather than between genotypes, but antigenic similarities were not as marked as genetic similarities. The detailed phylogenetic tree is shown in Fig 2, panel C.

### Antigenic cartography for *O*. *tsutsugamushi*

The antigenic maps for *O*. *tsutsugamushi* shown in Fig 2 plots the antigenic distance along the x- and y-axis using antigenic units. One antigenic unit corresponds to a two-fold difference in the patient serum titre. The predictive ability of the map was optimised when in three dimensions, however there was only a small improvement in two dimensions. When 20% of the titres were excluded, the average prediction error was 1.28 for 1D, 1.14 for 2D, 1.10 for 3D, 1.12 for 4D, 1.13 for 5D.

Although the number of strains in this dataset is not large, there were two main clusters of antigens: a Karp-like group and a Gilliam-like group. There was a single TA716-like strain, which was separate from the Karp-like and Gilliam-like groups. The positions of the sera in the antigenic map were not well clustered with the antigens (Fig 2, panel A). The sera against the TA716-like strain were indistinguishable from sera against Gilliam-like or Karp-like strains respectively. The sera from individuals infected with Gilliam-like strains generally had higher titres to the Gilliam-like strains than to the Karp-like strains, whereas the sera against Karp-like strains tended to be more broadly reactive (Fig 1).

One might expect paired homologous isolates and sera to be close to each other on the map. This is not seen in antigenic cartography of influenza, nor here. The average [±standard deviation] distance between a strain and its homologous serum was 1.8 [±1.1] antigenic units. The Gilliam-like strains tended to be closer to their homologous sera, and there was only a single representative of the TA716-like strains, limiting conclusions about their antigenic properties.

### Relationship between genotype and antigenic phenotype

Visual comparison of the phylogenetic tree, based on the amino acid sequences (Fig 2, panel C), demonstrate the clear distinction between Gilliam and Karp strains. The Gilliam-like strains, UT144, UT196, UT125 are identical, and are genetically close to FPW2016, FPW2049 and UT329. The Karp-like strains (see Table 1) are genetically close to each other. Since the Karp-like strains are similar genetically, it is expected that they would cluster together in the antigenic map when compared with the Gilliam-like strains that form a separate cluster.

The genetic map (Fig 2, panel B), recapitulates the results from the phylogenetic tree (Fig 2, panel C); the *O*. *tsutsugamushi* patient isolates can be grouped into three different types. There were not sufficient numbers of strains to reliably determine genetic correlates of antigenicity.

## Discussion

This study showed that there were three groups of antigenic reactivity corresponding to the genetic grouping of the Thai *O*. *tsutsugamushi* isolates in this study around three prototype strain genotypes; Karp, Gilliam and TA716 (Fig 2, panels A and B). As not all genetic differences contribute to antigenicity, the clustering in the antigenic maps is less distinct than observed in the genetic map.

The variable pattern of reactivity of sera from different individuals most likely has multiple causes, including differences in time since infection, variation between people in their response to infection, previous infections with homologous and/or heterologous Orientia strains and the antigenic phenotype of the infecting strain. This study investigated sera from acutely ill patients in an endemic area, but did not stratify the data by days of fever or determine possible strain-specific pre-exposure. In individuals infected with Karp-like strains, the serum titres, as measured by IFA, were often high to both Karp-like and Gilliam-like strains. In comparison, those infected with Gilliam-like strains mounted a response more focused on the Gilliam-like strains. This data does not illustrate cross-protection, but rather that anti-Karp sera reacted broadly within Karp and showed more cross-reactivity to Gilliam and TA716 clusters, while sera raised against Gilliam, remained very Gilliam-specific. This effect had been seen before where rabbit sera raised against some prototype strains was multi-specific [40]. Currently the human correlates of protection for scrub typhus are unknown, although phenotypic correlates have been described in a scrub typhus Rhesus macaque model [41]. The exact role of antibodies in protection against scrub typhus has not been fully determined yet, but neutralizing antibodies have been described in association with the 56kDa outer membrane protein, and that the majority of antibodies in the humoral response react against the 56kDa protein [23,25,26,42]. Although antigen-specific functional assays were not part of this study, our data suggests that a vaccine candidate eliciting a Karp-like strain antibody response or derivative would offer broader protection than a Gilliam-like response. However, for controlling this obligate intracellular pathogen, a multivalent, chimeric or T-cell based combination approach may be more appropriate [9,27]. Similarly, these techniques can be used to optimize strain choice in serological testing by determining the minimum set of antigens required to detect the majority of serological responses. The complexity of the serum response is part of the justification for using strain-specific monoclonal antibodies to antigenically characterise *O*. *tsutsugamushi*. However, this approach can mislead if the monoclonal antibodies do not focus on the same epitopes as whole human sera; it may be that whole sera are able to resolve more subtle differences within a serotype. Additionally, it may be valuable to further explore the complexity of the serological response, especially in humans.

A close relationship between the paired strain and homologous serum would be expected in the antigenic map given the strain-specific nature of the 56-kDa TSA and the specificity of the elicited immune responses. However, we found high average distances (expressed as antigenic units) between strains and their homologous sera. Often, the homologous antigen was not the maximum titre for a serum, which would tend to place that antigen away from its homologous serum (Table 3), a phenomenon which has been previously observed with influenza and dengue viruses [13,18]. The tension between the antigenic differences observed with the sera raised against the different strains contributed to the high distance between homologous strains and sera on the antigenic map. In a related manner, outlying sera (such as the one in the bottom left hand corner of Fig 2, panel A) have low titres to the antigens placed centrally and higher titres to the antigens towards the periphery of the map. Thus, the optimum placement of such a serum is out to one side of the map, often away from the homologous antigen. As an exact quantitation of antigen used on the IFA slides is challenging, a complete standardization was not achievable in this study, which may contribute to these patterns.

**Table 3:** Overview of all serological titres in this study.

*Sera*																								
*Isolates*		*UT76*	UT125	UT144	*UT150*	*UT167*	*UT169*	*UT176*	*UT177*	UT196	*UT213*	*UT219*	*UT221*	FPW2016	*FPW1038*	*FPW2031*	UT329	*UT332*	*UT336*	*UT395*	*UT418*	FPW2049	UT302	*UT316*
	***UT76***	**25600**	100	100	100	205600	1600	400	6400	6400	***25600***	***25600***	12800	800	12800	100	25600	25600	25600	12800	12800	12800	25600	12800
	**UT125**	12800	**25600**	25600	6400	102400	3200	800	6400	25600	***25600***	***25600***	12800	12800	12800	200	6400	6400	800	1600	25600	6400	25600	25600
	**UT144**	6400	25600	**25600**	1600	51200	1600	800	6400	25600	12800	12800	3200	6400	6400	100	6400	6400	800	3200	25600	12800	25600	12800
	***UT150***	6400	3200	6400	**400**	205600	3200	800	12800	400	3200	3200	1600	200	3200	100	1600	25600	800	1600	3200	800	1600	3200
	***UT167***	12800	3200	3200	6400	**205600**	1600	1600	***25600***	6400	***25600***	***25600***	6400	3200	25600	200	3200	25600	3200	25600	25600	12800	25600	25600
	***UT169***	25600	1600	1600	1600	205600	**6400**	1600	6400	1600	6400	12800	3200	400	6400	100	1600	25600	3200	25600	25600	12800	12800	25600
	***UT176***	12800	3200	3200	100	205600	1600	**400**	6400	400	800	3200	1600	400	12800	50	800	3200	800	1600	6400	3200	3200	3200
	***UT177***	12800	3200	1600	1600	205600	3200	800	**12800**	800	6400	3200	1600	800	12800	400	3200	25600	1600	12800	25600	25600	12800	12800
	**UT196**	25600	25600	6400	800	102400	1600	1600	800	**25600**	6400	***25600***	6400	12800	6400	50	6400	3200	400	800	25600	6400	25600	6400
	***UT213***	12800	12800	6400	3200	205600	3200	400	12800	1600	**12800**	3200	6400	800	3200	100	800	12800	1600	6400	12800	6400	1600	3200
	***UT219***	25600	6400	6400	3200	51200	***25600***	3200	***25600***	12800	12800	**12800**	12800	1600	12800	***800***	1600	12800	3200	25600	25600	12800	6400	12800
	***UT221***	25600	6400	3200	3200	102400	6400	1600	6400	1600	12800	3200	**25600**	800	12800	400	25600	25600	25600	25600	25600	12800	3200	12800
	**FPW2016**	25600	25600	25600	6400	205600	3200	3200	6400	25600	***25600***	***25600***	12800	**25600**	12800	***800***	25600	25600	6400	6400	12800	6400	25600	25600
	*FPW1038*	25600	6400	1600	1600	51200	6400	3200	12800	800	6400	3200	1600	800	**25600**	200	100	200	400	100	800	800	1600	6400
	***FPW2031***	6400	3200	6400	400	102400	3200	400	6400	800	12800	6400	6400	800	12800	**200**	800	6400	3200	3200	12800	3200	6400	12800
	**UT329**	25600	25600	25600	6400	102400	3200	1600	6400	25600	6400	12800	1600	12800	3200	50	**25600**	12800	800	1600	25600	6400	25600	6400
	***UT332***	25600	25600	25600	***12800***	205600	***25600***	***12800***	***25600***	800	***25600***	12800	3200	800	25600	200	3200	**25600**	1600	12800	25600	12800	6400	25600
	***UT336***	6400	1600	1600	200	102400	***25600***	800	12800	800	***25600***	6400	6400	800	12800	100	6400	25600	**25600**	12800	25600	12800	3200	25600
	***UT395***	12800	6400	6400	400	205600	***25600***	6400	***25600***	12800	12800	12800	12800	200	3200	400	12800	25600	12800	**25600**	25600	12800	1600	3200
	***UT418***	25600	3200	6400	800	205600	3200	800	12800	3200	6400	3200	6400	200	6400	50	1600	25600	3200	12800	**25600**	6400	3200	6400
	**FPW2049**	6400	6400	6400	800	102400	3200	800	1600	12800	6400	6400	3200	1600	3200	0	6400	12800	1600	800	6400	**25600**	6400	1600
	Maximum	25600	25600	25600	12800	205600	25600	12800	25600	25600	25600	25600	25600	25600	25600	800	25600	25600	25600	25600	25600	25600		

The strains and sera are formatted for comparisons to Fig 2; in the header rows the Karp strains are formatted in ***bold italics***, Gilliam strains in **bold normal**, TA716 strains in *regular italics*, and TA763 strains formatted in regular normal font.

Homologous titres in the diagonal are formatted in **bold**, and where the homologous titre is not the maximum, the greater than homologous titres are highlighted in ***bold italics***.

Antigenic cartography has typically been applied to antigenic datasets generated from laboratory animal sera with single-strain first infections [13]. Human serology is more complex as humans may have had prior or chronic infections and the time since contracting the disease is uncertain, and ideally first infection sera should be used to generate an antigenic map that is used as a guide to interpret the human serology [43]. An important caveat when interpreting these maps is that the true antigenic distances among *Orientia* isolates are not necessarily reflected, but rather how these particular patient sera relate to the antigens. As such, the antigenic relationships shown in this study may be influenced by the factors described above. Nevertheless, a map was constructed that had reasonable predictive power in two dimensions.

Previous work on other pathogens has generated maps in two or three dimensions; human influenza A/H3N2 and dengue are best described by an antigenic map with two dimensions [13,18]. For one dataset, plotting the map in more than one dimension over fitted the data, resulting in a map that did not perform as well at predicting missing titres despite increasing the number of parameters. However, a dataset with more isolates and sera was best fit in three dimensions suggesting that these additional titrations revealed more about the antigenic relationships. Furthermore, the map may change with the addition of more strains or sera of different antigenic types and from different times. Use of sera from a primary exposure may also affect dimensionality of the antigenic map. However, by analysing previously published data relating to influenza, we found that the most appropriate map generated from human sera was two-dimensional, in keeping with the two-dimensional map made using primary infection sera from ferrets and the same influenza viruses [43].

The apparent strain heterogeneity reflected by a 56-kDa TSA gene-based phylogenetic tree, was simplified upon dissection of the antigenicity of isolates and sera. Scrub typhus patient serum antibody responses were characterised by strong homologous, but weak heterologous antibody titres, with little evidence for cross-reactivity for Gilliam-like sera, but a broader response from some Karp-like sera. Antigenic cartography worked well with scrub typhus immunofluorescence titres. However, a large dataset comprising a broad selection of isolates, and inclusion of strain-specific reference sera raised in naïve animals, will enable further and more complete dissection of the antigenic relationships between Orientia strains and patient sera. This effort will require a network-based multinational collaborative approach.
